# Laser speckle contrast imaging identifies ischemic areas on gastric tube reconstructions following esophagectomy

**DOI:** 10.1097/MD.0000000000003875

**Published:** 2016-06-24

**Authors:** Dan M.J. Milstein, Can Ince, Suzanne S. Gisbertz, Kofi B. Boateng, Bart F. Geerts, Markus W. Hollmann, Mark I. van Berge Henegouwen, Denise P. Veelo

**Affiliations:** a Department of Oral and Maxillofacial Surgery, Academic Medical Center, University of Amsterdam, Amsterdam, The Netherlands; b Department of Translational Physiology, Academic Medical Center, University of Amsterdam, Amsterdam, The Netherlands; c Department of Surgery, Academic Medical Center, University of Amsterdam, Amsterdam, The Netherlands; d Department of Anesthesiology, Academic Medical Center, University of Amsterdam, Amsterdam, The Netherlands.

**Keywords:** blood perfusion, esophagectomy, gastric tube reconstruction, laser speckle contrast imaging

## Abstract

Gastric tube reconstruction (GTR) is a high-risk surgical procedure with substantial perioperative morbidity. Compromised arterial blood supply and venous congestion are believed to be the main etiologic factors associated with early and late anastomotic complications. Identifying low blood perfusion areas may provide information on the risk**s** of future anastomotic leakage and could be essential for improving surgical techniques. The aim of this study was to generate a method for gastric microvascular perfusion analysis using laser speckle contrast imaging (LSCI) and to test the hypothesis that LSCI is able to identify ischemic regions on GTRs.

Patients requiring elective laparoscopy-assisted GTR participated in this single-center observational investigation. A method for intraoperative evaluation of blood perfusion and postoperative analysis was generated and validated for reproducibility. Laser speckle measurements were performed at 3 different time pointes, baseline (devascularized) stomach (T0), after GTR (T1), and GTR at 20° reverse Trendelenburg (T2).

Blood perfusion analysis inter**-**rater reliability was high, with intraclass correlation coefficients for each time point approximating 1 (*P* < 0.0001). Baseline (T0) and GTR (T1) mean blood perfusion profiles were highest at the base of the stomach and then progressively declined towards significant ischemia at the most cranial point or anastomotic tip (*P* < 0.01). After GTR, a statistically significant improvement in mean blood perfusion was observed in the cranial gastric regions of interest (*P* < 0.05). A generalized significant decrease in mean blood perfusion was observed across all GTR regions of interest during 20° reverse Trendelenburg (*P* < 0.05).

It was feasible to implement LSCI intraoperatively to produce blood perfusion assessments on intact and reconstructed whole stomachs. The analytical design presented in this study resulted in good reproducibility of gastric perfusion measurements between different investigators. LSCI provides spatial and temporal information on the location of adequate tissue perfusion and may thus be an important aid in optimizing surgical and anesthesiological procedures for strategically selecting anastomotic site in patients undergoing esophagectomy with GTR.

## 1 Introduction

Gastric tube reconstruction (GTR) is a high-risk surgical procedure with substantial perioperative morbidity and a mortality rate of up to 5% in large centers.^[1]^ To improve long-term survival, patients often undergo neoadjuvant chemoradiation therapy. Although the majority of operations are performed as minimally invasive procedures, the incidence of complications associated with anastomosis such as leakage (4%–26%) and stenosis (12%–40%) remains high.^[2]^ During tumor resection with GTR, the left gastric, the short gastric, and the left gastroepiploic arteries are ligated. Only the right gastroepiploic artery at the greater curvature and the right gastric artery at the remains of the lesser curvature supply blood circulation to the newly formed gastric tube. The surgically compromised arterial blood supply and venous congestion after GTR are believed to be the main etiologic factor**s** associated with early (leakage) and late (stenosis) anastomotic complications. Moreover, the anastomosis is created from the fundus of the stomach where blood flow is most compromised. Irradiation injury, hypovolemia, vasopressor use, and the recommended semi-Fowler inclination postoperatively may further exacerbate gastric microcirculatory perfusion in the anastomotic region. Recognizing low-perfusion areas intraoperatively may identify patients at risk for anastomotic complications early and may provide a way of guiding surgical techniques for optimizing tissue perfusion. Currently, no such information is available to the surgical team before or during surgery.

Various measurements on the adequacy of microvascular blood flow in gastric mucosa perioperatively have been attempted with techniques such as reflection spectrophotometry, laser Doppler flowmetry (LDF), Doppler optical coherence tomography (DOCT), and laser-assisted fluorescence-dye angiography (LAA).^[3–6]^ However, despite best efforts, most investigations targeted endoluminal assessments of gastric mucosa perfusion and not epigastric blood perfusion. The available techniques do not enable measuring the adequacy of microcirculatory flow simultaneously in multiple regions of interest (ROIs) and the interpretation of the data can be difficult. Intraoperative real-time evaluation of epigastric microcirculatory perfusion may guide intraoperative judgment on fluid and vasomodulating therapy, and also surgical decisions on tissue excision and location of future anastomosis. Laser speckle contrast imaging (LSCI) is a noncontact near-infrared-based imaging system with high temporal and spatial resolution, providing an index of blood flow (flux) over large surface area**s**.^[7,8]^ Differing slightly from LDF principles, LSCI supersedes single-point blood flow information obtained by other instruments by yielding full-field perfusion maps of large anatomical areas with real-time 2D flux (blood flow) measurements based on speckle contrast analysis. With an extensive history of assessing skin microvascular reactivity, LSCI has been successfully used to measure blood perfusion on the cerebral cortex, liver, and renal cortex.^[9–12]^ Recently, the influence of elevated perfusion pressures on epigastric blood flow in a porcine model of GTR was investigated using LSCI.^[13]^ However, no human data are currently available regarding the feasibility of applying LSCI intraoperatively for assessments of GTR.

To evaluate the feasibility of intraoperative epigastric measurements with LSCI in humans, an assessment and comparison of tissue perfusion in different ROIs from intact stomach after ligation of the left gastric, left gastroepiploic, and short gastric vessels, and GTR was performed. The aims of this study were to generate and validate a reproducible method for epigastric perfusion analysis on human stomach and GTR and to evaluate whether perfusion parameters differed in different anatomic ROIs. We tested the hypothesis that LSCI was able to identify areas of diminished perfusion on GTR after esophagectomy.

## 2 Methods

The single-center observational study was performed between September 2014 and March 2015. The procedures and guidelines for this study were reviewed and approved by the Institutional Medical Ethics Committee of the Academic Medical Center of the University of Amsterdam (Ref. Nr. NL47619.018.14). All participants received detailed information about the study procedures, and signed informed consents were obtained. This investigation was performed in accordance with the principles established in the Declaration of Helsinki (Fortaleza, October 2013).

### 2.1 Study participants

Patients with esophageal carcinoma, referred to the Department of Surgery of the Academic Medical Center of the University of Amsterdam for elective minimally invasive 3-stage esophagectomy (McKeown procedure),^[14]^ were eligible for participation in this investigation. All patients received the same standardized anesthesia, surgical procedures, and intraoperative blood perfusion mapping of their partly devascularized stomach and GTR with LSCI. Patient demographic information such as weight, length, age, past medical history, medication use, American Society of Anesthesiologists (ASA) score, and treatment with chemoradiation were registered.

### 2.2 Perioperative and surgical procedures

#### 2.2.1 Anesthesia procedures

All patients were treated in the same operating theater kept at a constant 19 ± 1°C and rested in a supine position on the same operating table. A standardized anesthesia procedure was delivered to all patients by the same anesthesiologist (DPV). A thoracic epidural was inserted in a sitting position at the level of T 5–6 or 6–7. General anesthesia was induced with propofol (approximately) 2 to 3 mg/kg, 1.2 mg/kg of sufentanil, and 1 mg/kg rocuronium. The trachea was intubated and mechanical ventilation was started with pressure-regulated volume control of 6 to 8 mL/kg ideal body weight and an initial positive end-expiratory pressure (PEEP) of 5 cm H_2_O. Anesthesia was maintained with sevoflurane at a minimal alveolar concentration of 1. An arterial line and a right subclavian tri-lumen central line were inserted. The epidural catheter was loaded with bupivacaine 0.25% (10 mL) >15 minutes before incision. One hour later, a continuous infusion of bupivacaine 0.25% (0.1 mL/kg/hour) was started. In case of a failed epidural (i.e., epidural placement failure or malfunction during operation), 0.25 mg/kg bolus of ketamine and 0.6 to 0.8 μg/kg/hour sufentanil were infused continuously.

#### 2.2.2 Surgical procedures

In all patients, a 3-stage thoracolaparoscopic esophageal cardiac resection and GTR with cervical anastomosis was performed (McKeown).^[14]^ With the McKeown approach, the patient is oriented in a prone position directly after induction of anesthesia. During thoracoscopy, the esophagus is mobilized and an intrathoracic lymphadenectomy is performed (stations 2, 4, 5, 7, 8, and 9 according to the seventh edition of the AJCC).^[15]^ Next, the patient is rotated on the operating table back into a supine position. During laparoscopy, an abdominal lymphadenectomy is performed (stations 16–20 and lymph nodes in the hepatoduodenal ligament). The left gastric artery, part of the right gastric artery, the left gastroepiploic artery and the short gastric vessels, and, if present, the posterior gastric artery are ligated. The blood supply now comes from the right gastroepiploic artery, which supplies the greater curvature of the gastric tube from its origin (gastroduodenal artery) near the pylorus, and the right gastric artery, which supplies the remains of the lesser curvature. A 3 to 5 cm wide gastric tube was prepared and stapled longitudinally from the gastric angular notch towards the fundus outside the patient. After GTR, the anastomosis was secured in the neck with an end-to-end anastomosis. Intraoperatively, the location of the “watershed” area was identified and marked.

#### 2.2.3 Postoperative recovery

After surgery, general anesthesia was discontinued and the trachea was extubated in the operating theater if possible. Subsequently, the patient was transferred to the intensive care unit (ICU) (ASA > 3) or the postanesthesia care unit.

### 2.3 Monitoring

During the surgical procedure, heart rate (HR), diastolic, systolic, and mean arterial pressure (MAP), central venous pressure (CVP), peripheral capillary oxygen saturation (SpO_2_), end-tidal carbon dioxide (etCO_2_), insufflation pressure, and mean airway pressure were recorded. At every stage, the pressure reference was leveled to the tricuspid valve. MAP was maintained above 65 mm Hg or, in case of hypertension, within 30% of preoperative MAP. Norepinephrine was used to achieve these hemodynamic goals. HR was maintained < 100 bpm.

### 2.4 Full-field laser speckle contrast imaging

Intraoperative intact stomach and GTR surface blood perfusion assessments were obtained using a commercially available LSCI system (moorFLPI-1, Moor Instruments, Devon, UK) (Safety Standards: CE certified, class 1 Laser Product & EU Medical device directive classification: class IIa). Operated from a Windows-based computer system installed with the moorFLPI software (moorFLPI Measurement V3.0, Moor Instruments, Devon, UK), a divergent beam near-infrared 785-nm class 1 semiconductor laser diode was used to illuminate up to 1 mm depth on the surface of the tissue of interest. High-resolution (temporally-processed) speckle images were acquired using a 768 × 576-pixel grayscale charge-coupled device camera set to record a total of 5 frames for a duration of 50 seconds with a sampling interval of 10 seconds (0.1 Hz). The recorded image samples were converted to pseudo-color images coinciding with perfusion levels scaled from blue (low perfusion) to red (high perfusion).^[8,16,17]^ The exposure time coincided with 4 milliseconds and the temporal filter with 250 frames. The system's optics allow for adjustable zooming in a range between 0.6 × 0.8 cm (10 μm/pixel) and 9 × 12 cm at a working laser head to target (LH-T) distance of 15 to 45 cm. A 5 × 5-pixel window is used to calculate speckle contrast; maximum image resolution was 50 μm/pixel. The device focus and zoom dials were adjusted according to manufacturer's recommendations to achieve optimal image resolution in the field of view (FOV). The speckle lens is fitted with a tunable linear polarizing filter that was adjusted to diminish tissue surface reflections from the area of interest, that is, the moist exterior of the stomach surface. Before initiating measurements, the laser speckle instrument was calibrated according to the manufacturer's recommendations. For operational stability, the LSCI instrument was mounted onto an adjustable arm on top of a separate table and securely fixed to the tabletop using a sturdy clamping system.

### 2.5 Acquisition of blood perfusion data

All speckle data acquisition procedures and measurements were performed by the same investigator (DMJM) and according to the standardized procedures as described above. After dissecting and dividing the esophagus in the neck, the partially devascularized stomach was exteriorized and placed over the abdomen of the patient for perfusion measurements and GTR. A standardized setup was used for all perfusion data acquisitions: (1) full-organ surface blood perfusion imaging was obtained with the laser speckle lens aimed vertically and exactly perpendicular to the anterior organ surface wall with a LH-T distance of exactly 40 cm; (2) after optimal adjustment in focus, zoom, and polarizing filter settings, a sterile metric ruler was placed adjacent to the targeted organ in the speckle imager**'s** FOV **(**an important step for obtaining metric dimensions that were later used during postacquisition data processing); and (3) the watershed area between the left and right gastroepiploic artery was identified and marked by placing a piece of sterile surgical gauze with a blue lead line folded into a triangle with the tip and line pointing directly towards the middle of the watershed region, that is, the avascular area between the right and left gastroepiploic arteries. Since the sterile gauze used in the FOV is nonreflective, the tip of the triangle near the watershed was used to represent a zero perfusion reference point serving as an instrument signal calibration index during speckle measurements.

Laser speckle measurements were obtained in 50 seconds and were performed at 3 different time points: baseline (T0) perfusion imaging of the (partially devascularized) stomach was obtained with the operating table oriented flat (180°), 15 minutes later after GTR follow-up perfusion imaging with the operating table also oriented flat (180°) was obtained (T1), and finally 3 to 5 minutes later perfusion assessment of the GTR was obtained with the operating table oriented in a 20° reverse Trendelenburg configuration (T2).

### 2.6 Image analysis

Flux data analysis expressed in laser speckle perfusion units (LSPU) was performed offline using the moorFLPI analysis software package (moorFLPI Review V4.0, Moor Instruments, Devon, UK). A standardized systematic method for designing and analyzing flux perfusion was generated. To ascertain reproducibility, the metric ruler and sterile gauze tip indicating the watershed between the right and left gastroepiploic arteries needed to be present in the FOV in all measurements. With the metric ruler in the FOV, the distance between the watershed-to-cardiac notch (W-CN) was measured first in all stomachs from the center of the watershed region along the greater curvature up to the cardiac notch junction.

In the surgical procedure, 3 to 5 cm wide gastric tubes were constructed from the stomach. To maintain standardization 3-cm segments were selected along the length of the greater curvature of the stomach, starting from the center of the watershed and extending towards caudal and cranial, respectively. The analytical procedure was initiated by first creating a 3-cm line and a 3-cm diameter circle using the ruler in the FOV in the photo image. Next a new line was inserted across the cardiac notch crossing exactly the transition point from esophagus to stomach in the photo image. A second 3-cm line extending perpendicular from the center of the cardiac notch line towards the body of the stomach was inserted in the photo image. The tip of the second line delineated the border or limit of the most cranially placed ROI. Another 3-cm line extending from the exact center of the watershed towards the body of the stomach marked the border and separation between the first two ROIs (i.e., caudal right region (RR) 1 and cranial left region (LR) 1, respectively). Each ROI was created using 7 insertion points connected by lines using the polygon ROI mode from the analysis software. The remaining ROIs were subsequently inserted with 3-cm dimensions in both width and length following the greater stomach curvature, that is, RR2 (middle caudal region), RR3 (most vital caudal region), LR2 (middle cranial region), and LR3 (most ischemic cranial region) respectively. The 3-cm diameter circle was used as a guide to properly trace 3-cm lines for each ROI in the intact stomach; the circle contour followed the curving of the stomach and also provided a way of inserting 3-cm lines exactly perpendicular to a tangent line along the stomach curvature.

The 2 main criteria during the analyses were to include as many caudal (right) and cranial (left) ROIs as possible, extending from the center of the watershed along the intact stomach greater curvature in increments of 3 cm. Depending on the size of the stomach, right and left regions could go beyond RR3 and LR3, for example, RR4, RR5, LR4, and LR5. The other criterion was to carefully insert the ROIs exactly at the border of the stomach and not to include blood vessels and omental tissue extending inferiorly from the stomach's greater curvature. Finally, since the GTRs are all 3 to 5 cm wide, ROIs corresponding with 3-cm partitions (length) were easily inserted along the GTRs following the same analytical procedures as described above. Blinded from knowledge of each patient case, all speckle data analysis was separately analyzed by 2 independent examiners (DMJM and KB) according to the exact criteria and procedures described above.

### 2.7 Statistical analysis

#### 2.7.1 Sample size

Due to the novelty of using LSCI for intraoperative perfusion assessments of human GTR after esophagectomy, it is difficult to specify an a priori effect size. However, given the nature of the surgical procedure, we anticipated that there would be large effect sizes between measurements. For this proof-of-concept study, we performed a sample size calculation using paired-samples *t* test with an 80% power for detecting an LSPU effect size of 0.80 at a significance level of 0.05, resulting in a required sample size of 11 patients to detect GTR perfusional differences. This sample size is also supported by previous literature describing LSCI applications on human forearm skin, liver, and experimental research on gastric and hepatic microvascular perfusion studies.^[10,11,13,18]^

#### 2.7.2 Statistics

All data were checked for normality distribution according to the Shapiro–Wilk test. Intraclass correlation coefficients (ICC) and Bland–Altman analyses were performed to determine the extent of inter**-**rater reliability and mean percentage differences, respectively, for all LSPU flux datasets obtained from investigators 1 and 2. Repeated-measures analysis of variance (ANOVA), Wilcoxon, or Friedman test was used to compare ROI datasets at each time point, and a 2-way ANOVA was used to compare ROIs between the different measurements (T0, T1, and T2). All data analyses were performed using IBM SPSS statistics software package (IBM SPSS Statistics version 23, IBM Corp. Armonk, NY) and are presented as mean ± standard deviation (SD) unless stated otherwise; significant differences were detected when *P* < 0.05.

## 3 Results

A total of 11 patients were enrolled in this study. All surgical procedures were uneventful. A summary of all patient demographic information is presented in Table 1. Eighty-two per cent of the patients received chemoradiation therapy and 6 patients (67%) had the fundus of the stomach included in the field of irradiation. The mean W-CN distance in all intact stomachs measured along the greater curvature was 13.3 ± 2.7 cm. Postoperative anastomotic dehiscence occurred in 4 out of the 11 patients (36%). Mean W-CN distance was not significantly different between patients with postoperative anastomotic leakage and those without this complication (13 ± 3 vs 14 ± 3 cm, respectively).

**Table 1:**
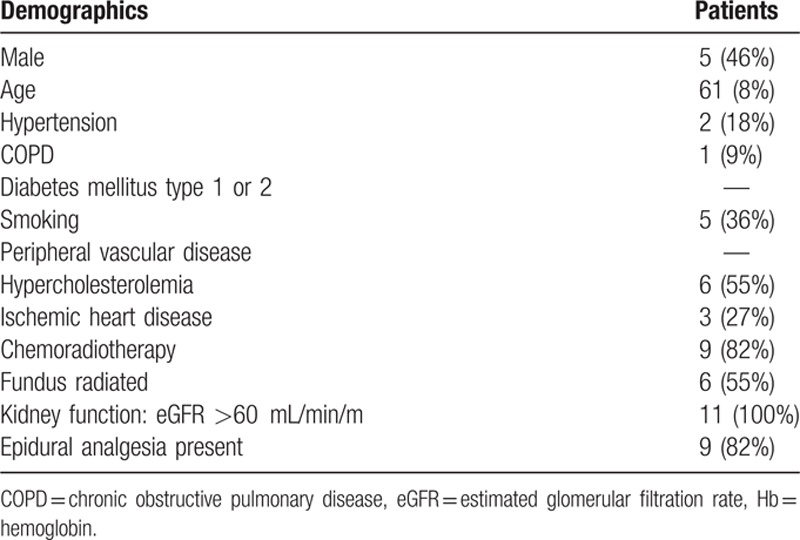
Patient demographic and clinical information.

A complete summary of all baseline hemodynamic parameters is presented in Table 2. There was a clinically small but significant difference in fluid balance (*P* < 0.001) and etCO_2_ (*P* = 0.005) between the different time points.

**Table 2:**
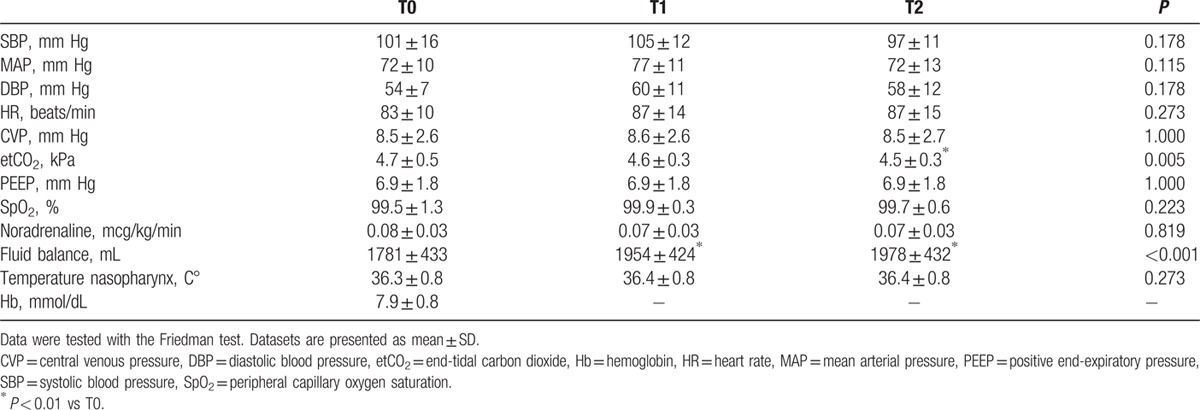
Hemodynamic and ventilation parameters corresponding with externalization of the stomach (T0), after gastric tube reconstruction (T1), and during 20° reverse Trendelenburg (T2).

### 3.1 Feasibility of measurements and validation of analysis

**Figure 1:**
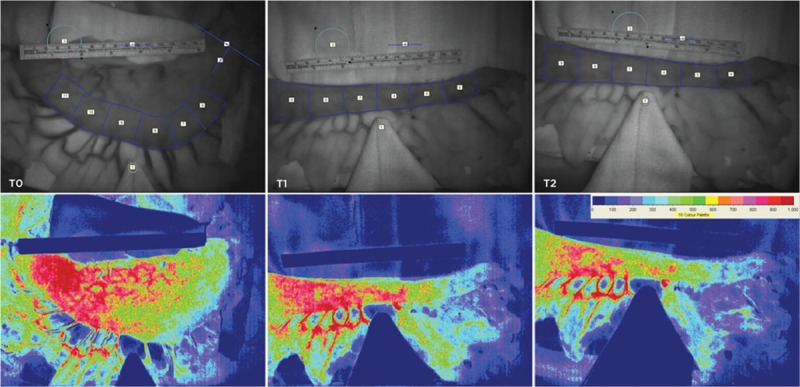
Frames obtained intraoperatively illustrating photo images (top row) of both the intact stomach and gastric tube reconstruction (GTR) analysis methodology with the targeted regions of interest. A matching sequence of typical laser speckle flux images (bottom row) is presented corresponding with the measurements of intact stomach (T0), after GTR (T1), and 20° reverse Trendelenburg GTR (T2).

**Figure 2:**
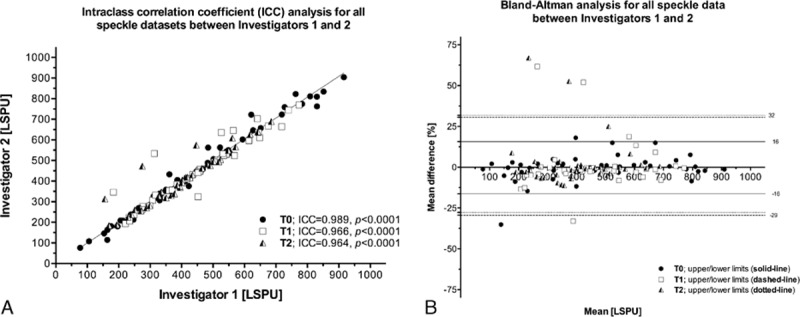
ICC (A) and Bland–Altman (B) analyses for all speckle datasets (i.e., T0, T1, and T2) between investigators 1 and 2. ICC = intraclass correlation coefficient.

Repeated intraoperative applications of LSCI successfully generated an overview or map of whole organ microvascular perfusion revealing ischemic and nonischemic regions instantaneously (Fig. 1). The setup of the LSCI in the theater was easy to perform and sterility of the operating field remained intact. The speckle imager produced high-quality images (total of 5 frames per time point) with excellent resolution for analysis offline. Inter**-**rater reliability in the acquired results (mean LSPU for each ROI and time point) was high, with an average ICC approximating 1 for all time points (*P* < 0.0001, respectively) (Fig. 2A). Bland–Altman plot shows low mean percentage differences between the 2 investigators (Fig. 2B).

### 3.2 Flux perfusion assessments gastric regions

**Figure 3:**
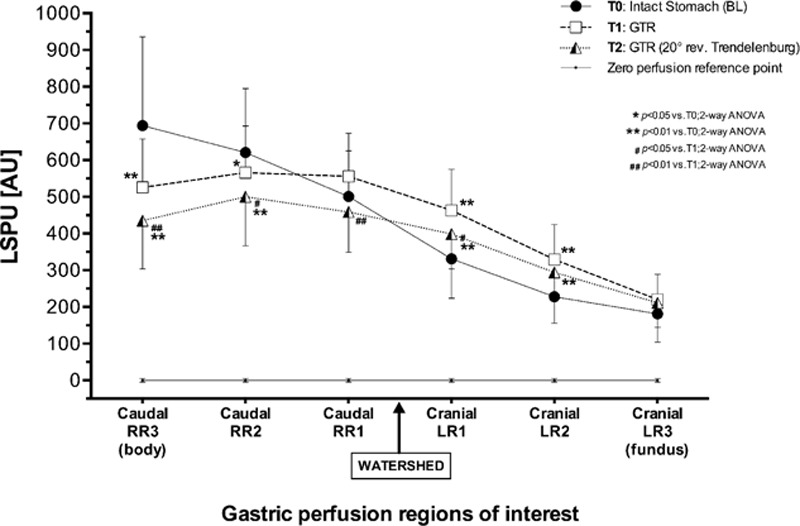
Graph summarizing 2-way ANOVA results of time points (T0, T1, and T2) for each region of interest (ROI) with laser speckle perfusion unit (LSPU) (flux data). ANOVA = analysis of variance.

Figure 3 and Table 3 present the mean LSPU for each ROI during T0 to T2. There was a significant decrease in mean LSPU from LR2 and LR3 versus the cranial regions (i.e., RR1, RR2, and RR3) across all time points (Table 3). At all 3 time points, mean LSPU at the base of the stomach and GTR (i.e., RR3) was highest (688 [237], 519 [126], and 434 [125], respectively) in comparison with the ischemic most cranial point or anastomotic tip (LR3) (175 [66], 207 ([64], and 202 [61], respectively**)** (*P* < 0.01). Interestingly, a significant improvement in gastric perfusion was observed after GTR (T1), with a rise in mean LSPU around the watershed region (RR1 and LR1) that continued into LR2 (before fundus) (*P* < 0.01) compared with T0. After inclining the patients at a 20° reverse Trendelenburg (T2), all the ROIs mean LSPU decreased significantly (*P* < 0.05), except in the regions that already had <350 LSPU, that is, near fundus (LR2) and fundus (LR3). There were no differences in flux parameters between patients who received radiotherapy on the area of the upper part of the gastric tube (the fundus) as compared with patients who did not receive radiotherapy.

**Table 3:**
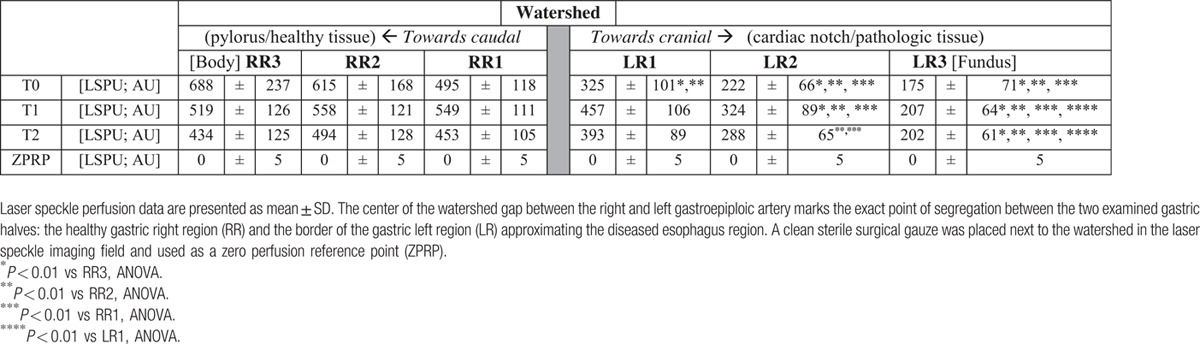
Overview of intraoperative gastric laser speckle perfusion unit (LSPU; arbitrary unit [AU]) datasets for all regions of interest before gastric reconstruction (T0) after gastric tube reconstruction (T1) and 20° reverse Trendelenburg.

## 4 Discussion

The study reports on the initial experiences with LSCI during esophagectomy with GTR. The results show that it is feasible to employ LSCI intraoperatively to generate an overview or mapping of intact, partially devascularized stomach, and gastric tube microvascular flow, at different time points during surgery. The results also demonstrate that the generated analytical approach was reproducible and able to adequately discern changes in tissue perfusion across different ROIs, especially around the area of the anastomosis between the left and right gastroepiploic arteries. Our data also suggest that this watershed region, the point of insertion of the gastroepiploic artery, differs between patients. This may be a factor in understanding risks involving postoperative anastomotic complications.

Earlier studies have presented various methods to measure the adequacy of microvascular blood flow in the gastric tube lumen perioperatively.^[3–6,19,20]^ As multiple small areas are needed to establish an overview of an entire organ's perfusion status inside a surgical field, most techniques are difficult to implement for clinical standards because of practical limitations and techniques like DOCT and LAA are much too invasive. Another known method used to visualize full-organ blood flow is through the use of indocyanine green fluorescence (ICG), but this has not been implemented as a standard.^[21]^ Recently using ICG, LAA was successfully employed intraoperatively in a series of esophagectomies with gastric pull-ups, and confirmed correlations between poor perfusion and anastomotic leaks versus good perfusion and anastomotic healing.^[6]^ Comparable with LSCI, LAA can be used intraoperatively to also obtain real-time perfusion datasets, yet LAA differs from LSCI as it is more invasive requiring ICG injections to achieve whole-organ imaging. LAA has as yet not been compared with LSCI. LSCI has been compared with other applicable techniques like LDF and DOCT.^[22,23]^ To the best of our knowledge, only 1 study has previously aimed LSCI to measure GTR in pigs.^[13]^ The speckle-derived flow data, as reported in the porcine study, show that perfusion is greatest at the base of the gastric tube and progressively decreases towards the anastomosis and gastric tube tip area; this was also shown in other (non-LSCI) studies.^[19,24]^ These anatomical observations match the results of our study on human gastric tubes, although comparison with our study is difficult, because no information was provided in their report regarding the exact location of the ROIs nor was an analytical strategy disclosed or validated. Interestingly, there was no improvement or change in blood flow in the porcine gastric tube regions after an attempt at raising MAP with a bolus of phenylephrine.^[13]^

**Figure 4:**
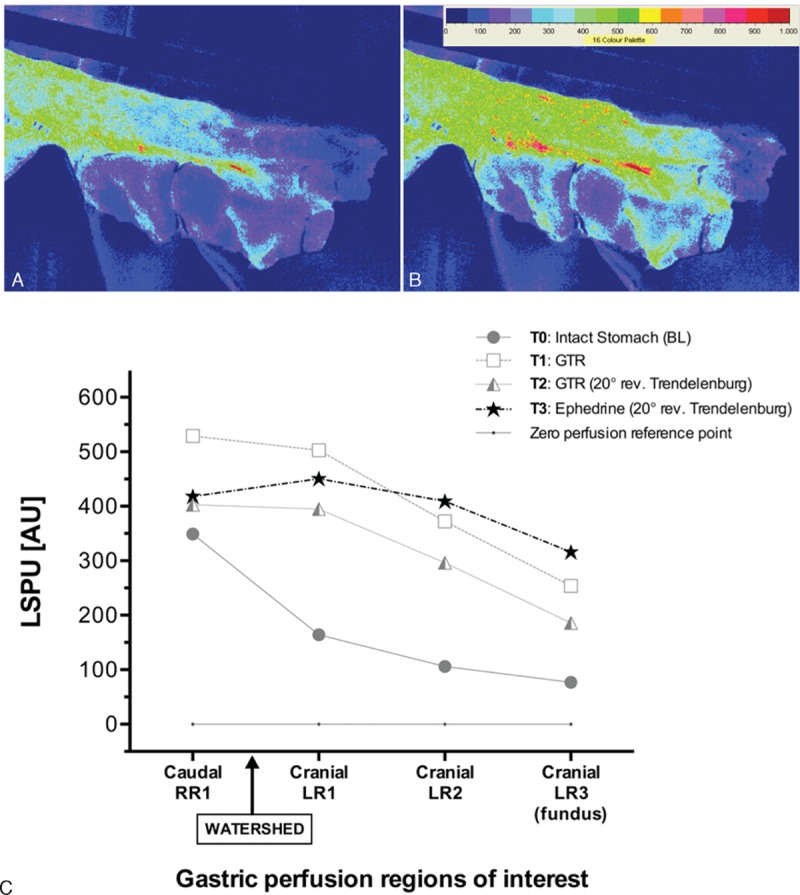
Supplementary intraoperative data from 1 patient illustrating the effects of administering ephedrine for treating low blood pressure on the most ischemic regions of the gastric tube during 20° reverse Trendelenburg. Flux perfusion measured by LSCI increased significantly from before (A) to after administering ephedrine (B) across all regions of interest (C), extending from the watershed towards the cranial (fundus) anastomotic tip. LSCI = laser speckle contract imaging.

Determining borders between highly vital and less vital (ischemic) tissue regions could help the surgeon intraoperatively to identify adequate locations for an anastomosis. Furthermore, full-field perfusion maps may support intraoperative fluid and vasomodulation more efficiently^[25–29]^; an example of this in 1 patient is presented in a supplementary graph (Fig. 4) showing the effects of administering ephedrine to treat low blood pressure, where flux perfusion measured by LSCI increased significantly. The use of local nitroglycerine improved microcirculation in some studies, but the use of vasoconstrictors aiming solely at improving perfusion pressure is doubtful.^[13,27,29]^ LSCI may provide valuable information regarding correlations between cardiac output and targeted visceral organ**s**. Although most patients received chemoradiation, only 6 patients had their fundus irradiated. It was not possible to obtain intact healthy human stomach speckle-based dataset**s** for comparison between devascularized and/or irradiated stomachs. It would have been a great advantage to obtain comparative data from healthy intact human stomach. We did not find any indications of affected microcirculatory flow in patients after radiation therapy on the fundus. This can be interpreted as either 1 of the 2 possibilities: (1) LSCI is not sensitive enough to detect more subtle changes, or (2) chemoradiation does not affect microcirculatory flow, and adverse outcome is caused by other complications such as changes in autonomic regulation of blood flow (divergence of flow), decreased regenerating capacity of cells, or long-term changes in tissue regeneration. Nonetheless, without healthy in situ stomach datasets for comparison, it remains difficult to interpret the impact of irradiation on fundus microvascular perfusion.

To ensure reproducibility, we based our systemic analysis on existing anatomical features; however, our study was not designed to interpret the clinical relevance of the watershed's anatomical location in relation to outcome. The location of the watershed (insertion of gastroepiploic artery) may determine the amount of ischemic tissue in the most cranial segment of the gastric tube, and may be a risk factor associated with anastomotic leakage. Previous studies by Miyazaki et al^[30]^ and Ikeda et al^[31]^ reported that anastomotic leakage was more common in patients with lower flow values. This study comprises a relatively small sample size for a clinical investigation, and larger studies are required to determine the value of this tool in clinical practice and whether surgical or anesthesiological adaptations based on LSCI measurements are possible and if they could potentially influence outcome.

Some important LSCI considerations and recommendations should be addressed. The systematic analysis of speckle microvascular perfusion maps was designed entirely based on innate anatomically available landmarks and the surgical procedure. Reproducibility validation for each dataset corresponding with the designated time points were confirmed, and comparisons between ROIs along the length of the stomach and gastric tube was possible. The method of analysis described in this study was reported in sufficient detail to provide an initiative platform for analytical standardization for future gastric perfusion-based research using LSCI. Moreover, the method of acquisition and data analysis may be interesting for other investigators or clinicians considering the design of studies involving the stomach and/or other visceral organs. To correct for system variations or faulty calibrations in laser speckle instruments, we recommend always including a true zero reference point to assess the consistency in flux recordings at that particular point in the FOV. Finally, although others report the possibility of achieving accurate perfusion measurements with LSCI from moving surfaces,^[32]^ data acquisitions should be obtained for best results from targets that are entirely static or motionless.

In conclusion, the results in this study demonstrate that it was feasible to implement LSCI intraoperatively to obtain blood perfusion assessments on intact and reconstructed whole stomachs. The analytical design presented and tested in this study yielded good reproducibility of gastric perfusion measurements between different investigators. Based on this first in-human gastric microvascular perfusion investigation using LSCI, perfusion profiles indicate that flux perfusion intensities are highest at the base or most caudal ROIs and progressively decline past the watershed towards the most cranial point or anastomotic tip. Accordingly, LSCI successfully conveyed spatial and temporal information about the location of adequate tissue perfusion and may thus be an important aid in ensuring surgical and anesthesiological procedures are optimized. The present results may have interesting implications for future determination of the location of the anastomosis for GTR. LSCI has potential clinical utility to gain; however, at this time, no intervention or modifications in surgical or anesthetic management will take place until further investigations indicate these benefits clearly.
