# Laser speckle contrast imaging identifies ischemic areas on gastric tube reconstructions following esophagectomy: Erratum

**DOI:** 10.1097/01.md.0000490344.77187.a5

**Published:** 2016-07-29

In the article “Laser speckle contrast imaging identifies ischemic areas on gastric tube reconstructions following esophagectomy”,^1^ which appeared in Volume 95, Issue 25 of *Medicine*, Tables 2 and 3 contained errors. The article has since been corrected online.
